# Can lifestyle factors explain racial and ethnic inequalities in all-cause mortality among US adults?

**DOI:** 10.1186/s12889-023-16178-6

**Published:** 2023-08-22

**Authors:** Klajdi Puka, Carolin Kilian, Yachen Zhu, Nina Mulia, Charlotte Buckley, Aurélie M. Lasserre, Jürgen Rehm, Charlotte Probst

**Affiliations:** 1 Institute for Mental Health Policy Research, Centre for Addiction and Mental Health (CAMH), Toronto, ON Canada; 2 Campbell Family Mental Health Research Institute, Centre for Addiction and Mental Health (CAMH), Toronto, ON Canada; 3 Department of Epidemiology and Biostatistics, Western University, London, ON Canada; 4 Alcohol Research Group, Public Health Institute, Emeryville, CA USA; 5 Department of Automatic Control and Systems Engineering, University of Sheffield, Sheffield, UK; 6 Addiction Medicine, Department of Psychiatry, Lausanne University Hospital, Lausanne, Switzerland; 7 Institute of Clinical Psychology and Psychotherapy, Technische Universität Dresden, Dresden, Germany; 8 Center for Interdisciplinary Addiction Research (ZIS), Department of Psychiatry and Psychotherapy, University Medical Center Hamburg-Eppendorf (UKE), Hamburg, Germany; 9 Program on Substance Abuse & WHO CC, Public Health Agency of Catalonia, Barcelona, Spain; 10 Dalla Lana School of Public Health, Department of Psychiatry, University of Toronto, Toronto, ON Canada; 11 I.M. Sechenov First Moscow State Medical University (Sechenov University), Moscow, Russian Federation; 12 Department of Psychiatry, University of Toronto, Toronto, ON Canada; 13 Heidelberg Institute of Global Health (HIGH), Medical Faculty and University Hospital, Heidelberg University, Heidelberg, Germany

**Keywords:** Race, Ethnicity, Health behaviors, Smoking, Alcohol, Physical activity, Obesity, BMI

## Abstract

**Background:**

Racial and ethnic inequalities in all-cause mortality exist, and individual-level lifestyle factors have been proposed to contribute to these inequalities. In this study, we evaluate the extent to which the association between race and ethnicity and all-cause mortality can be explained by differences in the exposure and vulnerability to harmful effects of different lifestyle factors.

**Methods:**

The 1997–2014 cross-sectional, annual US National Health Interview Survey (NHIS) linked to the 2015 National Death Index was used. NHIS reported on race and ethnicity (non-Hispanic White, non-Hispanic Black, and Hispanic/Latinx), lifestyle factors (alcohol use, smoking, body mass index, physical activity), and covariates (sex, age, education, marital status, survey year). Causal mediation using an additive hazard and marginal structural approach was used.

**Results:**

465,073 adults (18–85 years) were followed 8.9 years (SD: 5.3); 49,804 deaths were observed. Relative to White adults, Black adults experienced 21.7 (men; 95%CI: 19.9, 23.5) and 11.5 (women; 95%CI: 10.1, 12.9) *additional* deaths per 10,000 person-years whereas Hispanic/Latinx women experienced 9.3 (95%CI: 8.1, 10.5) *fewer* deaths per 10,000 person-years; no statistically significant differences were identified between White and Hispanic/Latinx men. Notably, these differences in mortality were partially explained by both differential exposure and differential vulnerability to the lifestyle factors among Black women, while different effects of individual lifestyle factors canceled each other out among Black men and Hispanic/Latinx women.

**Conclusions:**

Lifestyle factors provide some explanation for racial and ethnic inequalities in all-cause mortality. Greater attention to structural, life course, healthcare, and other factors is needed to understand determinants of inequalities in mortality and to advance health equity.

**Supplementary Information:**

The online version contains supplementary material available at 10.1186/s12889-023-16178-6.

## Background

Long-standing and stark racial and ethnic inequalities in health and mortality are widespread in the United States (US) [1, 2]. It is established that mortality rates among Black Americans are higher throughout most of the life course, relative to White Americans [2, 3]. In contrast, mortality rates among Hispanic/Latinx Americans are lower despite lower socioeconomic status (SES), on average, relative to White Americans [4]. In recent decades, research has focused on delineating the causes and etiology of racial and ethnic inequalities in mortality. A multitude of factors and pathways have been proposed and evaluated including societal influences (e.g., government policies) [5], environmental and occupational hazards (e.g., residential segregation) [6, 7], individual-level factors (e.g., SES, lifestyle factors, health insurance, and access to quality health care) [1, 2, 8–10], genetic factors [11], and potential biases in study designs, such as those related to selective migration (e.g., the salmon bias) [12, 13]. However, the complex and interrelated relationships and pathways in which these variables affect health and mortality have not been systematically evaluated and a large proportion of the observed racial and ethnic inequalities remains unexplained.

Lifestyle factors (such as smoking, alcohol use, physical inactivity, and obesity) are an important driver of inequalities in health, explaining, for example, more than two thirds of the association between SES and all-cause mortality [14, 15]. Although some evidence suggests that lifestyle factors are important in explaining racial and ethnic disparities [2, 3, 16, 17], no studies have used a comprehensive approach that evaluates multiple lifestyle factors together and their mediating and/or moderating effects. Evaluating multiple lifestyle factors is important as lifestyle factors may cluster together in distinct ways that vary by race and ethnicity [18, 19]. Understanding the mediating or moderating effects is essential in delineating potential mechanisms such as differential exposure, whereby health-promoting or unhealthy lifestyle factors are unevenly distributed across racial and ethnic groups (a mediation hypothesis), and differential vulnerability, whereby the same lifestyle factor can be more deleterious to specific racial and ethnic groups (a moderation hypothesis). Disentangling these two mechanisms is important given that unique policy implications can arise from them [20, 21].

**Fig. 1:**
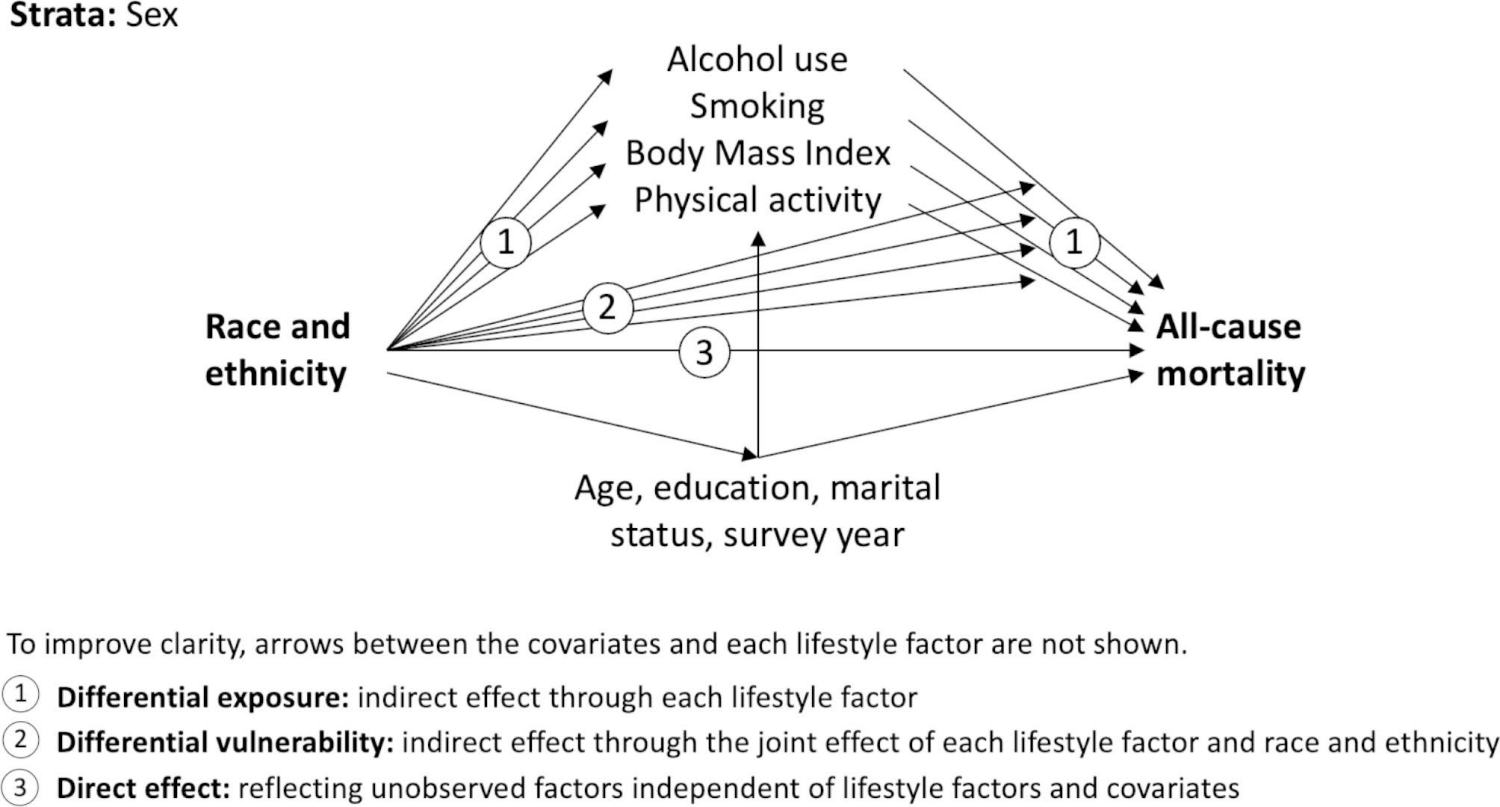
Diagram of the modelled direct and indirect relations between race and ethnicity, lifestyle factors, covariates, and all-cause mortality

Overall, the extent and means by which lifestyle factors might explain racial and ethnic disparities is largely unknown. Using a comprehensive model (Fig. 1) and a large cohort from the US, the current study aims to delineate the extent to which racial and ethnic differences in all-cause mortality can be explained by (i) *differential exposure* to lifestyle factors, and (ii) *differential vulnerability* to the harmful effects of each lifestyle factors across different race and ethnicity groups. The lifestyle factors considered were smoking, alcohol use, physical activity, and body mass index (BMI).

## Methods

### Participants

Data came from the National Health Interview Survey (NHIS) linked to the National Death Index (NDI) using probabilistic record matching [22]. NHIS is an annual, nationally representative, cross-sectional household survey of the civilian non-institutionalized US population (i.e., active duty members of the US military and individuals living in an institution such as residential care facilities or prisons were not sampled). NHIS utilized a complex, multistage sample design that involved stratification, clustering, and oversampling of specific population subgroups. Every year approximately 35,000 households are enrolled, from which one adult is randomly selected for a face-to-face interview. An annual assessment of all lifestyle factors in sufficient detail started in 1997, and NHIS data up to 2014 have been linked to the NDI. Therefore, this study included pooled NHIS data from 1997 to 2014. The NDI contains information on vital status, time of death, and time last presumed alive with follow-up to December 31, 2015. Our sample was comprised of the adults (ages ≥ 18 years) who were not missing data on the exposure, mediators, outcome, and covariates; those with complete and missing data were largely similar across a range of characteristics (Supplementary Table S1). Participants over 85 years of age at the time of NHIS administration were removed given that their exact age was not available through the public use data files.

### Measures

The outcome was time to all-cause mortality, operationalized as the time from the NHIS survey to death or last presumed alive. Race and ethnicity, the independent variable of interest, was self-reported and categorized as non-Hispanic White (reference category; henceforth White), non-Hispanic Black/African American (henceforth Black), and Hispanic/Latinx. We further distinguished all other non-Hispanic racial and ethnic groups (hereafter, non-Hispanic Other) for descriptive analyses, though sample size was too small for inclusion in the main analyses.

Participants’ report of the frequency and quantity of alcoholic beverage consumed in the past 12 months was converted to grams of pure alcohol consumed per day, assuming 14 g of pure alcohol per drink. Alcohol use was categorized according to the standards of the World Health Organization [23] and included: (1) never drinkers (no drinks in the past year and less than 12 drinks in any one year or entire life), (2) former drinkers (no drinks in the past year but have had at least 12 drinks in any one year), (3) category I (men: (0, 40] grams per day; women: (0, 20] grams per day; reference category), (4) category II (men: (40, 60] grams per day; women: (20,40] grams per day), (5) category III (men: >60 g per day; women: >40 g per day). With respect to smoking, participants were asked to report whether they (1) have smoked at least 100 cigarettes over their entire life, and (2) whether they currently smoked cigarettes. Smoking cigarettes was categorized as never smokers (reference category), former smokers, current someday smokers, and current everyday smokers. Based on self-reported height and weight, BMI was calculated and categorized according to current WHO guidelines as underweight (< 18.5 kg/m^2^), normal weight (18.5-24.99 kg/m^2^; reference category), pre-obesity (25-29.99 kg/m^2^) or obese (≥ 30 kg/m^2^) [24]. With respect to physical activity, participants reported how often and for how long they performed vigorous and light-moderate leisure-time physical activities of at least 10 min. No timeframe (e.g., over the past year, or past month) was specified. The length of moderate physical activity per week was calculated, assuming that 1 min of vigorous physical activity is equivalent to 2 min of moderate physical activity [25]. Physical activity was categorized as sedentary (0 min/week), somewhat active (< 150 min) or active (≥ 150 min; reference category), given the WHO recommendations of 150–300 min of moderate-intensity physical activity per week [26].

The covariates used in all models were age (continuous), sex, educational attainment, marital status, and survey year (continuous). Educational attainment was categorized as low (high school diploma or less), medium (some college but no bachelor’s degree), or high (bachelor’s degree or more), and was treated as a proxy for socioeconomic status; given its ubiquity in the extant literature, stability over time, and completeness of data (e.g., relative to income) in the NHIS. Marital status was a binary variable indicating whether the individual was married or living with partner.

### Statistical analyses

Causal mediation analysis using the marginal structural approach with Aalen’s additive hazard models was used, as described by Lange et al. [27–29]. Briefly, this flexible approach uses a counterfactual framework and allows for the direct parameterization of natural ‘direct’ and ‘indirect’ effects through multiple mediators and exposure-mediator interactions. The total effect of race and ethnicity on mortality was decomposed into three components (Fig. 1): (1) the average pure indirect effect through each mediator (indicating differential exposure), (2) the average indirect effect of the mediated interaction between race and ethnicity and each mediator (indicating differential vulnerability), and (3) the average ‘direct’ effect of race and ethnicity independent of mediators and covariates. The model simultaneously included all mediators (lifestyle factors: alcohol use, smoking, BMI, physical activity) and covariates (age, educational attainment, marital status, and survey year), and we fit separate models for men and women. Aalen’s additive hazard models have the advantage of directly estimating additive interactions (reflecting differential vulnerability), which are of greater importance (relative to multiplicative interactions) for public health [30].

All analyses were completed in R 4.1.3, using the *timereg* package (version 2.0.2) [31]; the statistical code is publicly available (see below). The *timereg* package does not allow for complex sampling designs and survey weights were not utilized given the analytical and computational complexity of the analyses.

In a sensitivity analysis, causal mediation models were repeated without education included as a covariate, recognizing that race and ethnicity are deeply tied to SES in the US [32], and prior research shows SES differences in effects of lifestyle factors on mortality [14, 15].

## Results

**Fig. 2:**
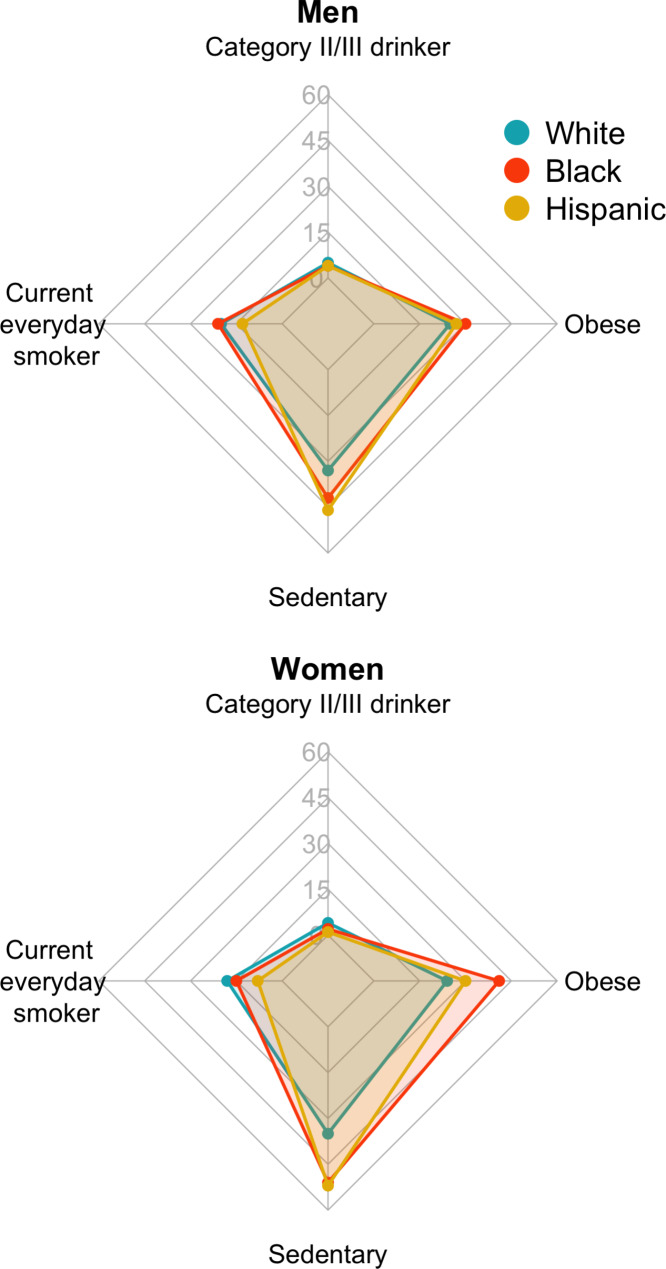
Visual representation of the prevalence (%, unweighted) of lifestyle factors posing higher health risks at baseline by sex and race and ethnicity

**Fig. 3:**
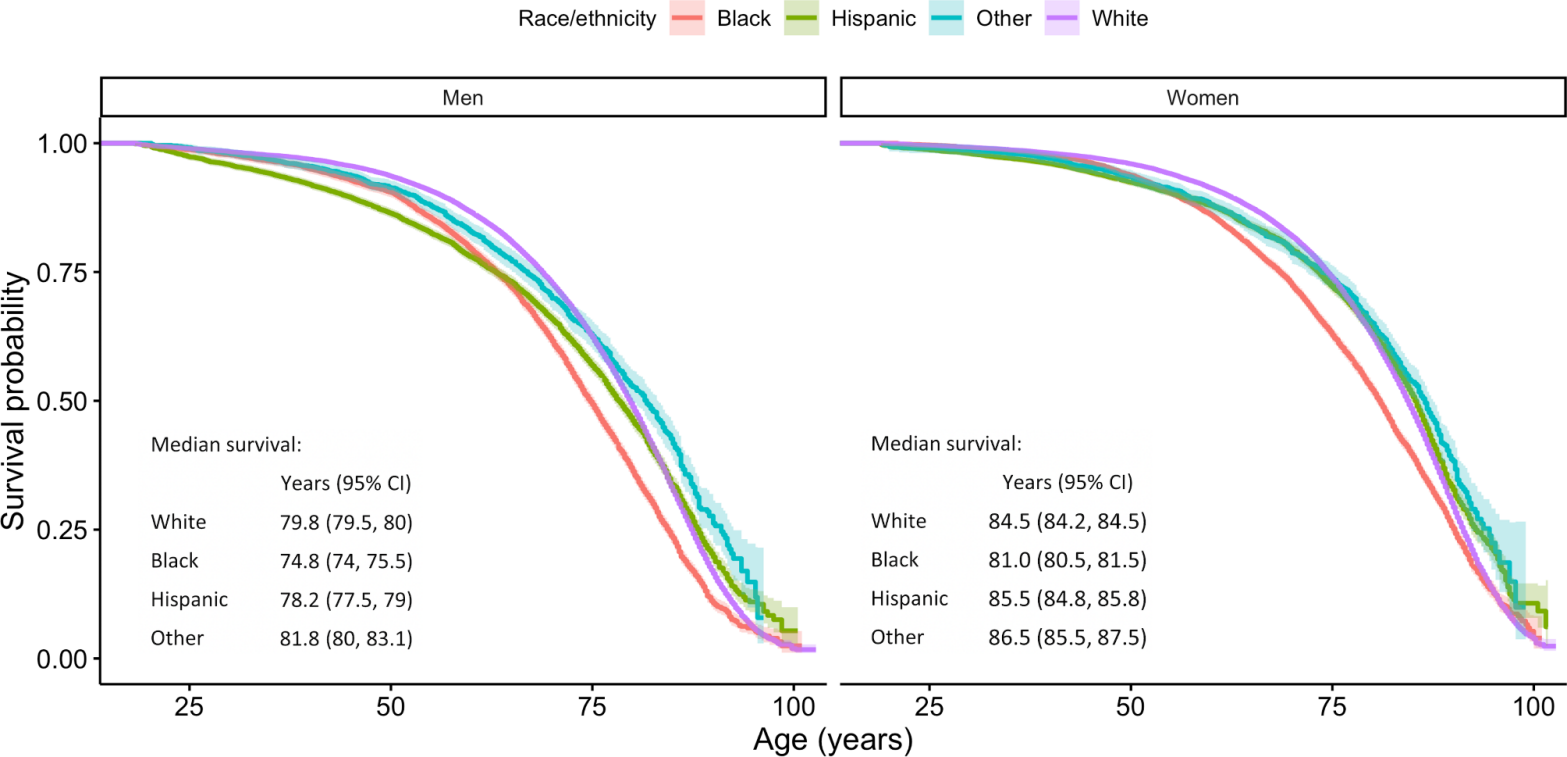
Survival probabilies stratified by sex and race and ethnicity

Participants were 465,073 adults (55% women, mean age 46.4 years [SD 17.3]), of whom 63% were non-Hispanic White, 15% non-Hispanic Black, 17% Hispanic/Latinx, and 5% non-Hispanic Other (of whom 12% were American Indian/American Natives, and 53% Asian and Pacific Islander Americans; see Table 1 for unweighted and Supplementary Table S2 for weighted data). Participants were followed an average of 8.9 years (SD 5.3) during which 24,296 and 25,508 deaths in men and women, respectively, were observed. At the time of survey completion, 22% of the men had never drunk alcohol, 50% had never smoked, 31% had a healthy weight, and 49% were physically active. In women, 39% had never drunk alcohol, 62% had never smoked, 42% had a healthy weight, and 40% were physically active. Relative to White adults, the prevalence of category II and III alcohol use and everyday smoking were lower among Black, Hispanic/Latinx, and non-Hispanic Other men and women (Fig. 2 for unweighted and Supplementary Figure S1 for weighted data). The opposite pattern was observed for obesity and sedentary physical activity, with a higher prevalence among Black and Hispanic/Latinx compared to White adults. Figure 3 presents the overall survival probability as a function of age, with the median survival probability being markedly lower in Black women (81 years, 95% confidence intervals [CI]: 80.5, 81.5) and men (74.8 years, 95%CI: 74.0, 75.5) than for other racial and ethnic groups (women: 84.5–86.5 years, men: 78.2–81.8 years).

**Table 1:** Participant characteristics (unweighted), stratified by sex and race/ethnicity

	Men				Women			
	White	Black	Hispanic	Other	White	Black	Hispanic	Other
Sample size, n	134,619	26,766	35,790	11,300	157,731	41,345	44,652	12,870
Age at survey, mean yrs (SD)	47.6 (17.0)	45.5 (16.4)	40.6 (15.4)	42.4 (16.0)	49.2 (17.8)	45.0 (16.9)	41.4 (15.9)	43.7 (16.7)
Follow-up, mean yrs (SD)	8.9 (5.3)	8.4 (5.1)	8.8 (5.2)	7.5 (4.9)	9.2 (5.3)	8.8 (5.2)	9.1 (5.2)	7.6 (5.0)
Person-years	1,203,789	224,185	315,056	85,213	1,447,070	364,681	407,088	97,830
All-cause deaths, n (%)	17,026 (13)	3,379 (13)	3,141 (9)	750 (7)	18,057 (11)	3,986 (10)	2,781 (6)	684 (5)
Alcohol use, %								
Never drinker	19	31	26	34	30	49	53	55
Former drinker	9	9	7	5	6	5	4	3
Category I (lowest)	67	56	63	59	60	43	42	40
Category II	3	2	2	1	3	1	1	1
Category III (highest)	3	2	2	1	1	1	0	0
Smoking, %								
Never smoker	46	53	59	60	55	67	78	80
Former smoker	30	19	20	20	23	13	11	9
Current some day smoker	4	7	8	5	4	5	4	3
Current everyday smoker	20	21	13	15	18	15	8	8
BMI, %								
Underweight	1	1	1	2	3	2	2	6
Healthy weight	31	30	27	48	46	27	36	59
Overweight	43	39	46	37	27	31	33	21
Obese	25	30	27	14	24	41	30	13
Physical activity, %								
Active	52	44	41	51	44	31	32	41
Somewhat active	16	14	13	17	21	18	16	22
Sedentary	33	42	46	32	35	51	52	38
Education %								
Highschool or less	39	54	68	27	40	51	66	33
Some college	30	30	21	25	32	33	24	25
Bachelor’s degree or more	31	16	11	47	28	16	11	42
Married/cohabitating, %	59	42	60	58	54	26	52	56
Born in United States, %	96	89	39	35	95	92	42	34

For weighted data, see Supplementary Table S2. py: person-years; SD: standard deviation; yrs: years

Table 2 presents the results of the causal mediation analyses, controlling for all covariates and lifestyle factors. Relative to White adults, Black adults experienced 21.7 (men; 95%CI 19.9, 23.5) and 11.5 (women; 95%CI 10.1, 12.9) *additional* deaths per 10,000 person-years, whereas Hispanic/Latinx women experienced 9.3 (95%CI 8.1, 10.5) *fewer* deaths per 10,000 person-years after adjusting for covariates. Mortality was similar among White and Hispanic/Latinx men, after adjusting for covariates.

**Table 2:** Results of causal mediation analyses, evaluating the extent to which the association between race and ethnicity with all-cause mortality was mediated by lifestyle factors

	Additional deaths per 10,000 py (95% CI)			
	**Black, Non-Hispanic Adults**		**Hispanic/Latinx Adults**	
	Men	Women	Men	Women
Effect of race/ethnicity (ref = White)	21.7 (19.9, 23.5)	11.5 (10.1, 12.9)	1.4 (0.0, 2.8)	-9.3 (-10.5, -8.1)
‘Direct’ effect of race/ethnicity (ref = White)	23.7 (21.8, 25.6)	17.1 (15.5, 18.7)	5.1 (3.6, 6.6)	-8.9 (-10.3, -7.5)
Net indirect effect of race/ethnicity (ref = White)	-2.0 (-4.9, 0.8)	-5.6 (-7.7, -3.4)	-3.6 (-5.7, -1.6)	-0.4 (-2.1, 1.3)
Alcohol use: differential exposure	1.4 (0.7, 2.0)	5.1 (4.5, 5.7)	-0.4 (-1.0, 0.2)	4.9 (4.3, 5.5)
Alcohol use: differential vulnerability	-2.6 (-4.2, -1.1)	-4.5 (-5.7, -3.3)	-0.7 (-1.9, 0.5)	-2.9 (-4.0, -1.9)
Smoking: differential exposure	-5.9 (-6.5, -5.3)	-10.4 (-11, -9.8)	-12 (-12.6, -11.4)	-17 (-17.6, -16.4)
Smoking: differential vulnerability	2.2 (0.7, 3.8)	1.0 (-0.2, 2.2)	6.4 (5.2, 7.6)	11.7 (10.6, 12.8)
BMI: differential exposure	0.5 (-0.1, 1.1)	0.8 (0.2, 1.4)	-1.9 (-2.5, -1.2)	-1.4 (-2.0, -0.8)
BMI: differential vulnerability	-1.4 (-2.9, 0.2)	-3.7 (-4.9, -2.5)	1.3 (0.1, 2.5)	1.8 (0.7, 2.8)
Physical inactivity: differential exposure	4.6 (3.9, 5.2)	7.8 (7.2, 8.4)	6.6 (6.0, 7.2)	7.5 (6.9, 8.1)
Physical inactivity: differential vulnerability	-0.7 (-2.2, 0.8)	-1.7 (-2.9, -0.5)	-2.9 (-4.1, -1.7)	-5.0 (-6.0, -3.9)

The models were stratified by sex and adjusted for age (as timescale), education, marital status, survey year, alcohol use (reference category: category I), smoking (reference category: never smoking), BMI (reference category: normal weight), and physical activity (reference category: physical active)

py: person years; CI: confidence interval

Table 2 further presents the effects of differential exposure and vulnerability to each lifestyle factor. The strongest effects were observed for smoking, finding that lower exposure to smoking resulted in 5.9 to 17.0 *fewer* deaths per 10,000 person years in Black and Hispanic/Latinx adults, relative to White adults (depending on the subgroup). However, Black men and Hispanic/Latinx adults were also more vulnerable to the adverse effects of smoking, which resulted in 2.2 to 11.7 *additional* deaths per 10,000 person years. The opposite pattern was observed for physical activity, finding that greater exposure to a sedentary lifestyle was associated with 4.6 to 7.8 *additional* deaths per 10,000 person years among Black and Hispanic/Latinx adults, relative to White adults, and that Black women and Hispanic/Latinx adults were less vulnerable to the adverse effects of physical inactivity, resulting in 1.7 to 5.0 *fewer* deaths per 10,000 person years. With respect to alcohol use, Hispanic/Latinx men were similar to White men. Among Black adults and Hispanic/Latinx women, exposure to alcohol use was associated with 1.4 to 5.1 *additional* deaths per 10,000 person-years, relative to White men and women. But this effect was partially offset by a greater resilience (differential vulnerability) to the adverse effects of alcohol use in these groups. Lastly, with respect to BMI, differential exposure and vulnerability effects were relatively small and offset each other.

Notably, the net indirect effect through lifestyle factors was not significant among Black men and Hispanic/Latinx women, and did not contribute overall to racial and ethnic inequalities in these racial and ethnic groups; different levels of physical activity were associated with *additional* deaths, whereas a lower prevalence of smoking was associated with *fewer* deaths. The results of the sensitivity analysis excluding education as covariate were consistent with our main analysis and did not change our conclusions (Supplementary Table S3). However, it is noteworthy that the total effect of race and ethnicity on mortality, as well as the net indirect effect were larger when not adjusting for education in our models.

## Discussion

The current study sought to evaluate the mechanism and extent to which lifestyle factors contribute to racial and ethnic inequalities in mortality among US adults. Specifically, we examined whether these inequalities can be explained by indirect effects through differential exposure and differential vulnerability to harmful effects of different lifestyle factors.

First, and consistent with the extant literature [2–4], we found that relative to White adults, mortality rates were higher for Black men and women, and lower for Hispanic/Latinx women. Our key finding and the novel contribution of this study was that mechanisms of differential exposure and vulnerability to lifestyle factors help to explain the disparity in mortality rates between White and Black women, and the equivalent mortality rates of White and Hispanic/Latinx men. This was, however, not the case for Black men and Hispanic/Latinx women. In other words, lifestyle factors cannot explain the observed racial and ethnic inequalities in all-cause mortality in the latter groups. This is because the net indirect effect of race and ethnicity through differential exposure and vulnerability to lifestyle factors did not contribute to the observed inequalities among Black men and Hispanic/Latinx women given individual indirect effects canceled each other out. Specifically, *additional* deaths among Black men and Hispanic/Latinx women were attributed to a higher exposure to sedentary physical activity, while a lower prevalence of smoking resulted in *fewer* deaths, relative to White men and women. This finding that particularly highlights the differential exposure to different lifestyle factors across racial and ethnic groups is consistent with past studies [3, 16, 33, 34]. As for differential vulnerability, the observed effects were small overall and might be linked to unmeasured mortality risk factors associated with different lifestyle factors. For example, prior research has found that White alcohol users are at increased risk for alcohol-impaired driving [35] and for continuing to drink heavily following a diagnosis of a serious health condition compared to other racial and ethnic groups [36]. Such factors could result in White men and women being more vulnerable to the detrimental health effects of alcohol, as observed in our findings. The results of the current study help to advance the extant literature through our use of a comprehensive model to decompose the effects of differential exposure and vulnerability. Our results suggest that public health interventions targeting physical inactivity among Black and Hispanic/Latinx adults are important. However, targeting lifestyle factors alone, without consideration of more fundamental forces, such as poverty, structural racism, and limited opportunity [37], will not likely improve racial and ethnic disparities in mortality observed for Black men and women.

Our findings for the somewhat limited role of lifestyle factors in explaining racial and ethnic inequalities in mortality stand in contrast to research on socioeconomic disparities in mortality, which report that the latter inequalities are largely attributed to the net indirect effect of lifestyle factors [14, 15]. This difference in findings may be because lifestyle factors putting individuals at higher health risks were found to cluster among low SES groups [14, 15], in contrast to our finding of a lower prevalence of smoking among Black and Hispanic/Latinx adults which resulted in a relative protective effect. Taken together, these findings have important public health implications in highlighting that socioeconomic and racial and ethnic inequalities in mortality in the US may arise in unique ways (e.g., racial residential segregation is likely more relevant to the Black-White mortality gap) and likely require distinctive intervention approaches. Even so, past studies have shown that SES is an important mediator of racial and ethnic inequalities in mortality [3, 16, 38], and reducing socioeconomic inequalities in mortality potentially by targeting the root causes of socioeconomic health inequalities may in turn also reduce racial and ethnic disparities. The role of SES in racial and ethnic inequalities in mortality was further stressed in our sensitivity analysis, in which we have repeated the causal mediation models without adjusting for education, resulting in a higher total and net indirect effect of race and ethnicity on all-cause mortality in Black and Hispanic/Latinx adults. Lower racial and ethnic inequalities in mortality when adjusting for education appear plausible, as education is an important predictor of disease onset and progression, and racial and ethnic disparities in education exist [39]. This finding further suggests an intersectional dimension of both factors.

In interpreting the results presented above, limitations should be considered. First, the choice of covariates is important given that causal mediation models assume no unmeasured confounders for the exposure-outcome, exposure-mediator, and mediator-outcome relationships, and no mediator-outcome confounders caused by the exposure. Mediators are also assumed to have no causal effect on each other. Residual confounding by unmeasured risk factors in our analyses is possible. In particular, chronic health conditions and diet quality were not taken into account and might vary across racial and ethnic groups. Second, because the data arose from participants’ self-report from a single time point, reporting bias and changes in lifestyle factors over time may have introduced misclassification and underestimated the association between lifestyle factors and mortality. Third, the exclusion of the institutionalized population in the NHIS may have led to an underestimation of racial and ethnic inequalities in mortality, as well as the indirect effects of lifestyle factors, as racial and ethnic minorities are likely to be overrepresented in some of these populations [40]. Moreover, lifestyle factors linked to increased mortality risks, such as category II and III alcohol use, are expected to be higher in the institutionalized than in the non-institutionalized population [41]. Fourth, we were unable to account for migration dynamics, which may have impacted on survival probabilities, as movements vary across racial and ethnic groups. However, it needs to be acknowledged that the US has a positive net migration rate [42] and prior research on the impact of migration on lower mortality among Hispanics residing in the US concluded that out-migration appears to have only little impact on their mortality advantages [13]. Lastly, given the analytical and computational complexity of the analyses we could not account for the complex survey design of the NHIS, which may have affected estimates of the indirect effects through differential exposure in particular. However, comparing the prevalence of lifestyle factors in the weighted (Table 1) and unweighted data (Supplementary Table S2), only minor differences are observed, suggesting that not accounting for the complex survey design is unlikely to have substantially influenced our results concerning differential exposure to lifestyle factors. Given this analytical and computational complexity and due to sample size considerations, our analyses also did not separate US- and foreign-born Hispanic/Latinx adults, which is an important differentiator given that foreign born status and acclimatization in the US are important factors contributing to Hispanic/Latinx’s mortality [2, 12]. Similarly, a more detailed disaggregation and analysis of the non-Hispanic Other group was not possible.

Overall, our study of multiple lifestyle factors demonstrates that their net effect helps to explain some portion of the observed racial and ethnic inequalities in all-cause mortality. While differential exposure and vulnerability to multiple lifestyle factors contributed to the disparity in Black women’s all-cause mortality, in other groups, indirect effects of individual lifestyle factors canceled each other out. Importantly, lifestyle factors do not develop in isolation but are a product of more fundamental forces associated with structural and social determinants of health [37]. Future work should endeavour to understand the differential exposure and vulnerability effects of other factors potentially underlying racial and ethnic inequalities in mortality, including societal factors, environmental and occupational hazards, and other individual-level factors not necessarily related to lifestyle such as exposure to stress and resilience.

### Electronic supplementary material

Below is the link to the electronic supplementary material.

Supplementary Material 1

## Data Availability

The data underlying this article are available from National Center for Health Statistics; the dataset was derived from sources in the public domain: https://www.cdc.gov/nchs/nhis/1997-2018.htm and https://www.cdc.gov/nchs/data-linkage/mortality-public.htm. The statistical code is available at https://github.com/yachenz1/SIMAH_Ethnicity_x_Lifestyle.
