# Supplementary material for: Can lifestyle factors explain racial and ethnic inequalities in all-cause mortality among US adults?
Source: BMC Public Health. 2023 Aug 22;23:1591. doi: 10.1186/s12889-023-16178-6 (PMC10464312; doi:10.1186/s12889-023-16178-6)
Supplement: Supplementary file 1 — Supplementary Material 1 [file 12889_2023_16178_MOESM1_ESM.docx]

**Lifestyle risk factors and racial and ethnic inequalities in mortality among US adults**

Klajdi Puka, Carolin Kilian, Yachen Zhu, Nina Mulia, Charlotte Buckley, Aurélie M. Lasserre,

Jürgen Rehm, and Charlotte Probst

**Supplementary File**

Contents

[Supplementary Table S1. Participant characteristics at time of survey completion, comparing those with complete and missing data. 2](#_Toc134688124)

[Supplementary Table S2. Participant characteristics (weighted), stratified by sex and race/ethnicity. 3](#_Toc134688125)

[Supplementary Table S3. Results of causal mediation analyses, without educational attainment as a covariate. 4](#_Toc134688126)

[Supplementary Figure S1. Visual representation of the prevalence (%, weighted) of lifestyle factors posing higher health risks at baseline by sex and race and ethnicity. 5](#_Toc134688127)

# Supplementary Table S1. Participant characteristics at time of survey completion, comparing those with complete and missing data.

|  | Men | | |  | Women | | |
| --- | --- | --- | --- | --- | --- | --- | --- |
|  | Complete data | Missing data | SMD |  | Complete data | Missing data | SMD |
| Sample size, n | 208,475 | 30,168 |  |  | 256,598 | 44,312 |  |
| Age at survey, mean yrs (SD) | 45.9 (16.8) | 45.0 (16.5) | 0.055 |  | 46.9 (17.6) | 47.3 (17.0) | 0.023 |
| Alcohol use, % |  |  | 0.121 |  |  |  | 0.133 |
| Never drinker | 22 | 27 |  |  | 38 | 45 |  |
| Former drinker | 8 | 8 |  |  | 5 | 5 |  |
| Category I (lowest) | 65 | 60 |  |  | 53 | 48 |  |
| Category II | 3 | 2 |  |  | 2 | 2 |  |
| Category III (highest) | 2 | 2 |  |  | 1 | 1 |  |
| Smoking, % |  |  | 0.065 |  |  |  | 0.031 |
| Never smoker | 50 | 50 |  |  | 62 | 64 |  |
| Former smoker | 26 | 23 |  |  | 18 | 18 |  |
| Current some day smoker | 5 | 6 |  |  | 4 | 4 |  |
| Current everyday smoker | 19 | 20 |  |  | 15 | 15 |  |
| BMI, % |  |  | 0.122 |  |  |  | 0.147 |
| Underweight | 1 | 1 |  |  | 3 | 3 |  |
| Healthy weight | 31 | 35 |  |  | 42 | 47 |  |
| Overweight | 43 | 44 |  |  | 29 | 28 |  |
| Obese | 25 | 21 |  |  | 27 | 21 |  |
| Physical activity, % |  |  | 0.299 |  |  |  | 0.205 |
| Active | 49 | 37 |  |  | 40 | 32 |  |
| Somewhat active | 15 | 12 |  |  | 20 | 17 |  |
| Sedentary | 36 | 51 |  |  | 41 | 51 |  |
| Education % |  |  | 0.104 |  |  |  | 0.066 |
| Highschool or less | 45 | 50 |  |  | 46 | 49 |  |
| Some college | 28 | 26 |  |  | 30 | 29 |  |
| Bachelor’s degree or more | 27 | 23 |  |  | 24 | 22 |  |
| Married/cohabitating, % | 57 | 55 | 0.041 |  | 49 | 50 | 0.023 |

py: person-years; SD: standard deviation; yrs: years.

SMD: standardized mean difference, with values of 0.2, 0.5, and 0.8 interpretable as small, medium, and large effect sizes, respectively.

# Supplementary Table S2. Participant characteristics (weighted), stratified by sex and race/ethnicity.

|  | Men | | | |  | Women | | | |
| --- | --- | --- | --- | --- | --- | --- | --- | --- | --- |
|  | White | Black | Hispanic | Other |  | White | Black | Hispanic | Other |
| Sample size, n | 67,294,176 | 10,220,568 | 12,767,668 | 4,796,436 |  | 69,358,008 | 12,425,076 | 12,266,709 | 5,049,583 |
| Age at survey, mean yrs (SD) | 46.1 (16.8) | 41.7 (16.0) | 38.8 (14.8) | 41.9 (15.6) |  | 47.1 (17.5) | 42.8 (16.5) | 40.3 (15.7) | 42.4 (15.8) |
| Follow-up, mean yrs (SD) | 8.9 (5.0) | 8.6 (5.0) | 8.2 (4.9) | 8.2 (4.9) |  | 9.0 (5.0) | 8.8 (5.0) | 8.4 (5.0) | 8.1 (4.9) |
| All-cause deaths, % | 11 | 10 | 7 | 7 |  | 9 | 8 | 5 | 5 |
| Alcohol use, % |  |  |  |  |  |  |  |  |  |
| Never drinker | 19 | 33 | 26 | 35 |  | 30 | 50 | 52 | 57 |
| Former drinker | 8 | 7 | 6 | 5 |  | 5 | 5 | 4 | 3 |
| Category I (lowest) | 68 | 56 | 64 | 58 |  | 61 | 43 | 43 | 38 |
| Category II | 3 | 2 | 2 | 1 |  | 3 | 1 | 1 | 1 |
| Category III (highest) | 2 | 2 | 2 | 1 |  | 1 | 1 | 0 | 0 |
| Smoking, % |  |  |  |  |  |  |  |  |  |
| Never smoker | 47 | 58 | 62 | 60 |  | 57 | 70 | 80 | 82 |
| Former smoker | 29 | 17 | 19 | 19 |  | 22 | 12 | 10 | 8 |
| Current some day smoker | 4 | 7 | 8 | 5 |  | 4 | 4 | 4 | 3 |
| Current everyday smoker | 20 | 19 | 12 | 16 |  | 18 | 14 | 7 | 8 |
| BMI, % |  |  |  |  |  |  |  |  |  |
| Underweight | 1 | 1 | 1 | 2 |  | 3 | 2 | 2 | 6 |
| Healthy weight | 30 | 30 | 27 | 47 |  | 47 | 28 | 36 | 60 |
| Overweight | 43 | 39 | 45 | 36 |  | 27 | 30 | 33 | 22 |
| Obese | 26 | 30 | 28 | 15 |  | 23 | 40 | 29 | 13 |
| Physical activity, % |  |  |  |  |  |  |  |  |  |
| Active | 52 | 46 | 42 | 49 |  | 45 | 31 | 34 | 40 |
| Somewhat active | 16 | 14 | 14 | 18 |  | 21 | 18 | 17 | 22 |
| Sedentary | 32 | 41 | 45 | 33 |  | 34 | 50 | 49 | 38 |
| Education % |  |  |  |  |  |  |  |  |  |
| Highschool or less | 40 | 53 | 66 | 29 |  | 39 | 49 | 64 | 33 |
| Some college | 30 | 31 | 22 | 26 |  | 32 | 34 | 25 | 25 |
| Bachelor’s degree or more | 31 | 16 | 11 | 46 |  | 28 | 17 | 12 | 42 |
| Married/cohabitating, % | 68 | 51 | 65 | 66 |  | 65 | 36 | 61 | 66 |
| Born in United States, % | 100 | 90 | 40 | 30 |  | 100 | 90 | 40 | 30 |

# Supplementary Table S3. Results of causal mediation analyses, without educational attainment as a covariate.

|  | Additional deaths per 10,000 py (95% CI) | | | |
| --- | --- | --- | --- | --- |
|  | **Black, Non-Hispanic Adults** | | **Hispanic/Latinx Adults** | |
|  | Men | Women | Men | Women |
| Total effect of race/ethnicity (ref=White) | 32.8 (30.9, 34.6) | 16.0 (14.6, 17.5) | 17.6 (16.2, 19.0) | -0.5 (-1.7, 0.8) |
| ‘Direct’ effect of race/ethnicity (ref=White) | 27.4 (25.5, 29.3) | 15.3 (13.7, 16.9) | 13.3 (11.9, 14.7) | -7 (-8.4, -5.7) |
| Indirect effect of race/ethnicity (ref=White) | 5.4 (2.5, 8.2) | 0.7 (-1.4, 2.8) | 4.3 (2.3, 6.2) | 6.6 (4.8, 8.3) |
| Alcohol use: differential exposure | 3.9 (3.2, 4.6) | 7.4 (6.7, 8.0) | 2.6 (2.0, 3.2) | 8.3 (7.7, 9.0) |
| Alcohol use: differential vulnerability | -4.2 (-5.7, -2.6) | -5.5 (-6.7, -4.3) | -2.4 (-3.6, -1.2) | -3.8 (-4.9, -2.8) |
| Smoking: differential exposure | -3.1 (-3.8, -2.5) | -9.7 (-10.3, -9.0) | -9.3 (-10, -8.7) | -17 (-17.7, -16.3) |
| Smoking: differential vulnerability | 1.9 (0.4, 3.5) | 2.3 (1.0, 3.5) | 5.9 (4.8, 7.1) | 13 (11.9, 14.1) |
| BMI: differential exposure | 1.2 (0.5, 1.8) | 1.6 (0.9, 2.3) | -1.3 (-1.9, -0.6) | -0.9 (-1.5, -0.2) |
| BMI: differential vulnerability | -1.7 (-3.2, -0.2) | -3.9 (-5.1, -2.6) | 1.2 (0.1, 2.4) | 2.0 (1.0, 3.1) |
| Physical inactivity: differential exposure | 9.0 (8.3, 9.6) | 10.6 (9.9, 11.2) | 14.1 (13.4, 14.8) | 12.3 (11.6, 13) |
| Physical inactivity: differential vulnerability | -1.7 (-3.2, -0.2) | -2.1 (-3.3, -0.9) | -6.6 (-7.8, -5.4) | -7.4 (-8.5, -6.3) |

The models were stratified by sex and adjusted for age (as timescale), marital status, survey year, alcohol use, smoking, BMI, and physical activity. py: person years; CI: confidence interval


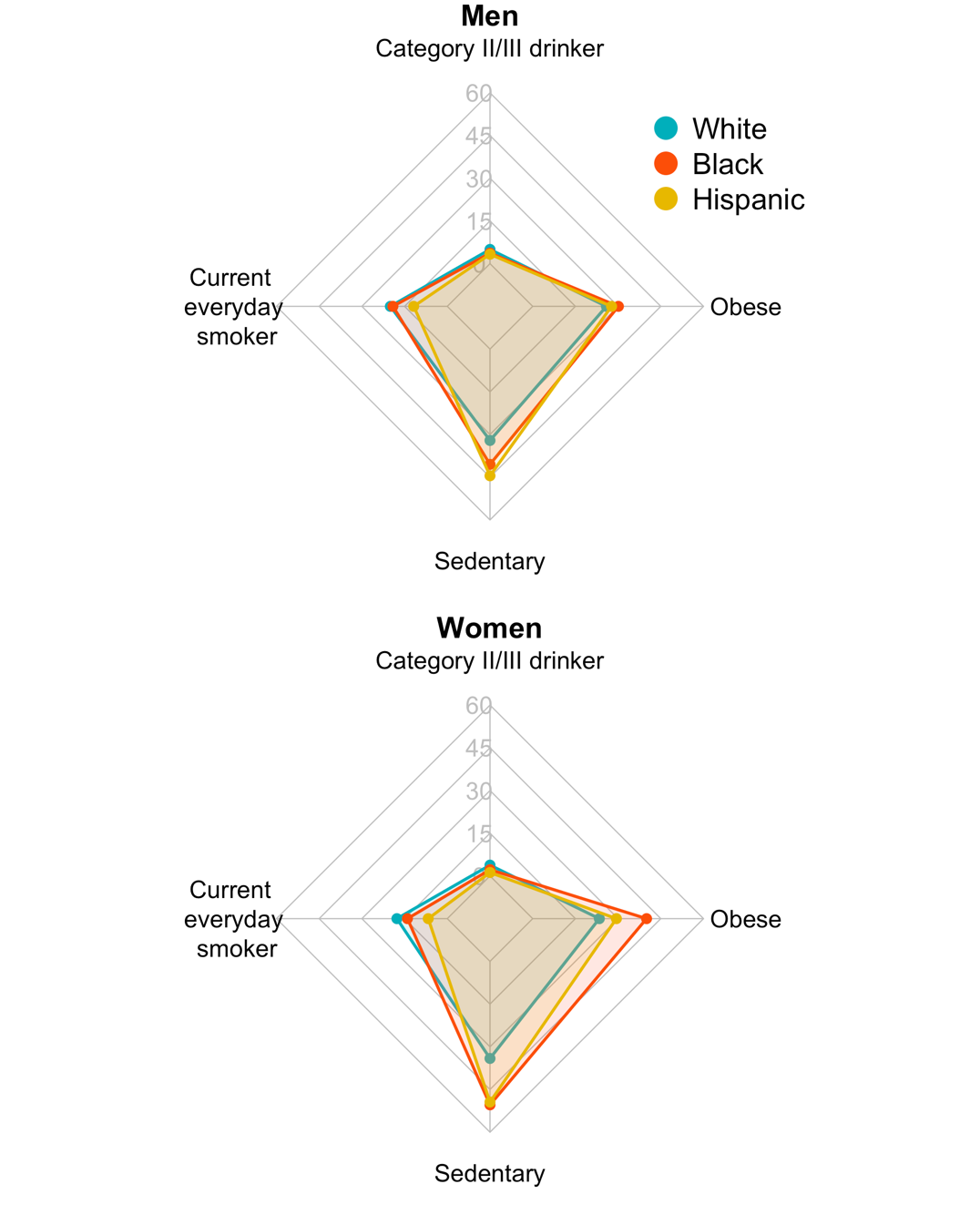


# Supplementary Figure S1. Visual representation of the prevalence (%, weighted) of lifestyle factors posing higher health risks at baseline by sex and race and ethnicity.
